# Nitrogen limitation as a driver of genome size evolution in a group of karst plants

**DOI:** 10.1038/srep11636

**Published:** 2015-06-25

**Authors:** Ming Kang, Jing Wang, Hongwen Huang

**Affiliations:** 1 Key Laboratory of Plant Resources Conservation and Sustainable Utilization, South China Botanical Garden, Chinese Academy of Sciences, Guangzhou, China

## Abstract

Genome size is of fundamental biological importance with significance in predicting structural and functional attributes of organisms. Although abundant evidence has shown that the genome size can be largely explained by differential proliferation and removal of non-coding DNA of the genome, the evolutionary and ecological basis of genome size variation remains poorly understood. Nitrogen (N) and phosphorus (P) are essential elements of DNA and protein building blocks, yet often subject to environmental limitation in natural ecosystems. Using phylogenetic comparative methods, we test this hypothesis by determining whether leaf N and P availability affects genome sizes in 99 species of *Primulina* (Gesneriaceae), a group of soil specialists adapted to limestone karst environment in south China. We find that genome sizes in *Primulina* are strongly positively correlated with plant N content, but the correlation with plant P content is not significant when phylogeny history was taken into account. This study shows for the first time that N limitation might have been a plausible driver of genome size variation in a group of plants. We propose that competition for nitrogen nutrient between DNA synthesis and cellular functions is a possible mechanism for genome size evolution in *Primulina* under N-limitation.

Genome size, measured as the haploid nuclear DNA content (1C-value), is of fundamental biological importance with implications for predicting structural and functional attributes of organisms^1^,^2^,^3^. As one of most variable traits of biodiversity, C-value varies over 2400-fold among species of angiosperm plants and has been a longstanding puzzle in evolutionary biology^4^,^5^. Although abundant evidence has shown that the C-value enigma can be largely explained by differential proliferation and removal of non-coding DNA of the genome^6^,^7^,^8^, the evolutionary and ecological basis for genome size variation remains poorly understood and highly controversial^9^,^10^,^11^. Nitrogen (N) and phosphorus (P) are essential components for production of nucleic acids and protein, but they are often subjected to ecological limitation in most natural ecosystems. Thus, a biological stoichiometry approach should enable us to unravel the connections between genome and the ecological interactions among organisms and their environments.

Recent biological stoichiometry studies have shown that environmental N-limitation can affect nucleotide composition of an organismal genome^12^. For example, the genome-wide comparison of nitrogen element between wild and crop plants revealed that the wild *Arabidopsis thaliana* use less nitrogen in their genomes and proteins than crops, which was attributed to ecological nitrogen limitation in *A. thaliana*^13^. Thus, it is conceivable that nutrient limitation may not only influence the molecular composition, but also the size, of the genome (i.e. the amount of DNA) of organisms in resource-limited habitats. No explicit tests of this hypothesis exist, although a few studies support some of the main assumptions. Small genome sizes are frequently reported in carnivorous plants, whose growth is generally both N and P co-limited^14^. For example, genome sequencing revealed a minute genome size of 82 Mb for the carnivorous plant *Utricularia gibba* living in P-poor freshwater habitats^15^. The recently proposed ‘growth rate-genome size-nutrient limitation’ hypothesis (GGNH) predicted that the reallocation of P from DNA to RNA under the selection favoring rapid growth in nutrient-limited environments could lead to genome downsizing in eukaryotes^16^. This hypothesis received experimental supports from a few studies on zooplanktonic groups such as *Caenis* spp. and *Daphnia* spp.^17^,^18^,^19^, but no explicit experimental data from plants available to test the hypothesis. In fact, the relationship between genome size and growth rate in plants has remained controversial^20^,^21^. Nevertheless, a long-term fertilization experiment in local plant communities has shown that soil P availability may play a role in selection of plants with different genome sizes^22^. A recent comparative analysis revealed a tendency of genome size expansion from non-geophytes to their geophytic relatives, for which the storage organs were assumed to be serviced as nutrition reserves and therefore make geophytes relatively independent of the nutrient availability in their environment^23^. Yet, no direct evidence of nitrogen limitation on genome size variation has been reported in plants.

The limestone karsts in Southeast Asia harbor a highly diverse and unique biota, and have long been regarded as “natural laboratories” for ecological and evolutionary research addressing natural selection^24^. Soils in the karst area are typically shallow and characterized by low soil water content, periodic water deficiency, and N limitation^25^, which exert strong selective forces on plant evolution, resulting in remarkably high species richness and endemism in the region. *Primulina*, a genus of the African violet family Gesneriaceae, is a monophyletic group comprising more than 140 species of perennials that are widely distributed throughout the karst regions of China and adjacent countries of Southeast Asia^26^. The distribution of the genus spans a wide latitudinal range (18 °N–31 °N) and most species occur only in calcareous soil associated habitats developing from limestone bedrock (Fig. 1). A recent large-scale analysis of genome size for 101 species revealed variation up to 2.27-fold differences of the 2C DNA content among species although chromosome numbers were constant (2n = 36)^27^. The rich species diversity and soil-habitat specialization together with a wide range in genome sizes make *Primulina* an excellent model for studying the impact of nutrient limitation on genome size variation in terrestrial plants.

Phylogeny-based comparative analysis of the genome size in *Primulina* detected significant positive relationships between genome size and specific leaf area (SLA) as well as between genome size and latitudes, indicating adaptive evolution of genome size in this genus^27^. We hypothesize that the adaptive genome size in *Primulina* is likely driven by selection pressure of nutrient limitation towards “efficient” genomes for survival in response to harsh karst habitats. We test this hypothesis by exploring the relationship between genome size and leaf C/N/P content and their respective ratios for 99 species of *Primulina* with consideration of their phylogenetic history. We find that genome sizes in *Primulina* are strongly positively correlated with leaf N content. This study represents, to the best of our knowledge, the first test of the hypothesis that nutrient limitation drives genome size evolution in a group of phylogenetically closely related species.

## Results

Leaf N and P concentrations and N : P ratio varied greatly across species, with a range of 5.38–38.38 mg g^−1^ for N, 0.46–5.12 mg g^−1^ for P and 3.82–41.24 for N : P ratio (Table 1). The average values were 14.65 mg g^−1^ for N, 1.45 mg g^−1^ for P and 11.46 mg g^−1^ for N : P. Leaf N concentration and C : N ratio exhibited a significant amount of phylogenetic signal (λ = 0.759–0.776, *P* < 0.0001; Table 1), implying strong phylogenetic dependence of these traits. The estimates of λ for leaf P and C : P were only marginally significant (*P* < 0.048), whereas leaf C and N : P showed no significant phylogenetic signal (*P* > 0.556).

**Figure 2:**
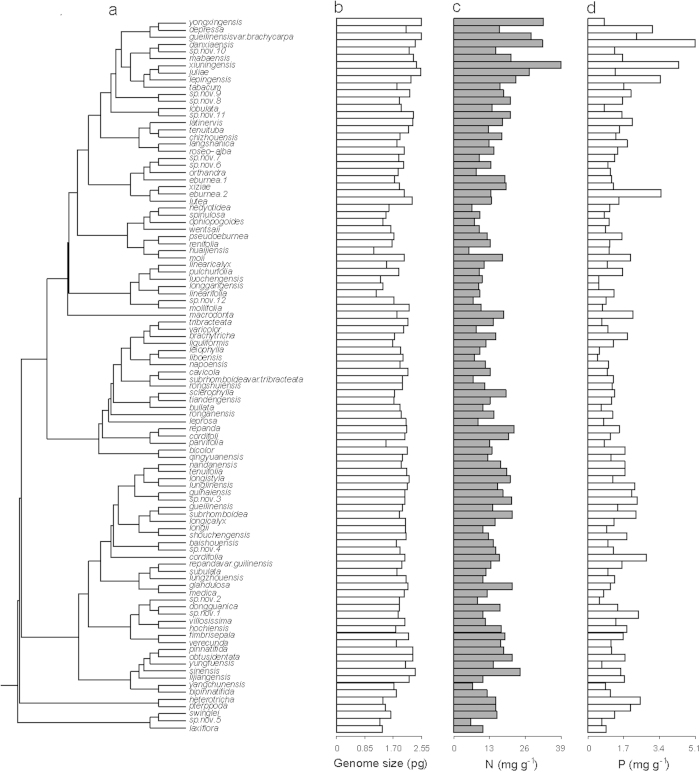
A phylogenetic tree of 99 species of *Primulina* (**a**) with their genome size (**b**), leaf N (**c**) and P (**d**) concentrations. The phylogenetic tree was adopted from Kang *et al.* (2014).

**Figure 3:**
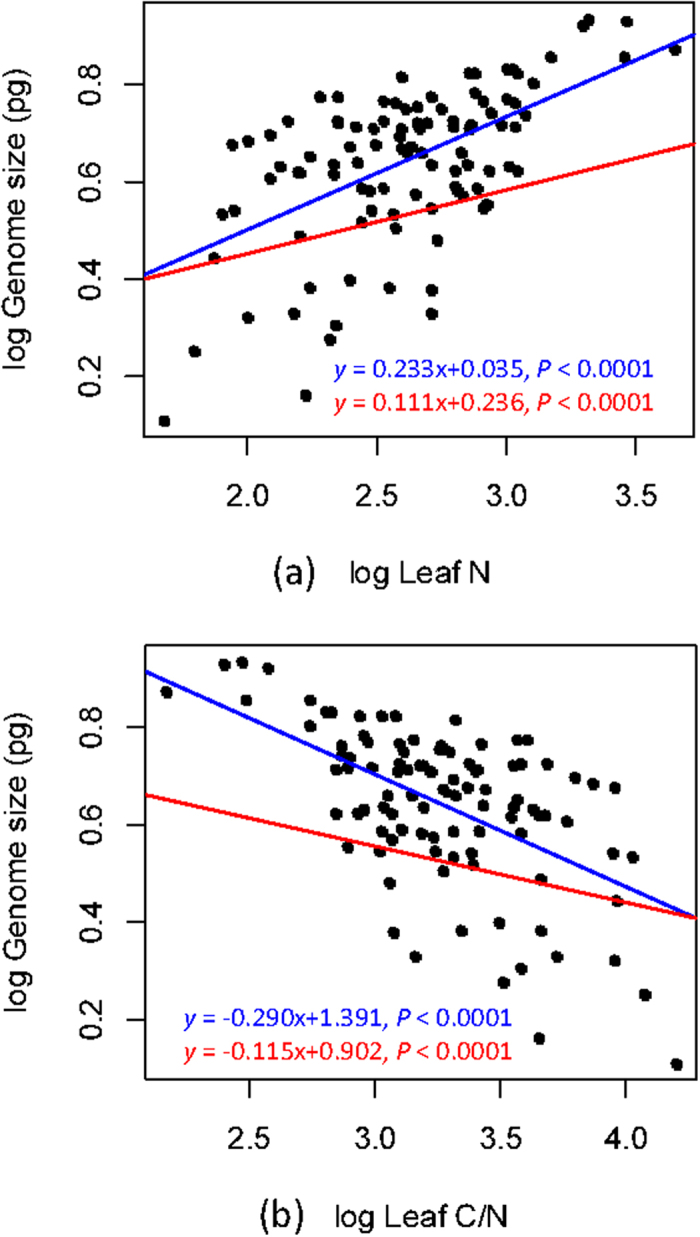
Genome size (2C DNA content) in relation to (**a**) leaf N concentration and (**b**) ratio of C:N under regression models of ordinary least squares (OLS; blue) and phylogenetic generalized least squares (PGLS; red).

Figure 2 shows the phylogenetic tree of *Primulina* species with genome size and leaf concentrations of N and P. Genome size was significantly and positively correlated with leaf N concentration and negatively correlated with leaf C : N ratio under both phylogenetic generalized least squares (PGLS) and ordinary least-square (OLS, i.e. nonphylogenetic regression) regressions (Fig. 3, Table S2). Although the PGLS yielded a weaker explanatory power (adjusted *R*^2^ = 0.098–0.120) than OLS (adjusted *R*^2^ = 0.290–0.299) (Fig. 3, Table S2), the likelihood ratio tests (LRT) showed that the PGLS model fit the data better than the OLS model in both comparisons (Table S2). Because the leaf C content is similar between species (Table 1), the negative correlation of genome size with leaf C : N ratio is likely to be driven by leaf N. Under the PGLS model, leaf N was significantly correlated with latitudinal climate variation, but more tightly with temperature-related than precipitation-related variables (Table S3). Although we found significant relationships between genome size and leaf P and leaf C : P ratios under the OLS model, such relationships were driven by phylogenetic non-independence and disappeared under PGLS model (Table S2).

## Discussion

The evolution of genome sizes is probably due to multiple interdependent mechanisms^28^. Nutrient limitation has been proposed as one such mechanism for plants and invertebrates that need high demands of N and P for growth^16^. The average leaf N concentration in *Primulina* (14.65 mg g^−1^) was much lower than that reported in a study of 1900 plant species across China (22.3 mg g^−1^)^29^ and usually well below concentrations that were thought to limit growth^30^. However, leaf P concentration fell into the general range reported in other plants (1.68 mg g^−1^)^29^, and thus resulting in relatively low mean N : P ratio (11.46) (Table 1). According to the criterion of N limitation at N < 20 mg g^−1^ and N : P < 14^30^,^31^, the growth of *Primulina* appears to be N limited. The strong correlation of genome size with leaf N concentration suggests that N limitation may have been a driving force of genome size variation in *Primulina*.

To date, the only two experimental studies have found contrasting influence of P availability on plant genome size^22^,^32^. A recent study in the Mediterranean region revealed that P availability in soil or plant has not affected genome size stability^32^, while a long-term (60 years) fertilization experiment shown that soil P availability may play a role in selection of plants with different genome sizes^22^. It is most likely that variation in sensitivity to P availability among lineages may result in taxon-specific effect on genome size. In our study, we investigated the relationships between genome size and nutrient availability with two different methods: the nonphylogenetic OLS and phylogenetic generalized least-squares (PGLS). Our likelihood ratio tests (LRT) showed that the PGLS model fits the data better than the OLS model. Under PGLS model, there was no significant correlation between plant P concentration and genome size, suggesting P availability is not a determining factor for genome size variation in *Primulina*.

It is widely recognized that the elemental demands of N and P for producing nucleic acid are highly costly^33^. Assuming a 1:1 ratio for purines and pyrimidines, N and P comprise on average approximately 39% and 9% of nucleic acid mass, respectively^12^. Therefore, the availability of N and P could have a direct influence on nucleic acid synthesis. The ‘growth rate-genome size-nutrient limitation’ hypothesis (GGNH)^16^ suggests that the reallocation of P (and eventually N) from DNA to RNA synthesis could be under selection in nutrient-limited environments. This hypothesis predicts that species with small genome sizes would have faster growth rates, however this hypothesis is based on a few observations in animals^16^, so far no experimental test has been attempted in plants. Our field investigations found that *Primulina* species with small genome sizes usually occur in more harsh southern regions and show slow growth grate. Consistent with the ‘stress resistance syndrome’ (SRS)^34^, these observations in *Primulina* may represent an adaptive strategy enabling lineages to persist in the harsh karst environment. *Primulina* usually grows on shallow, nutrient poor and limestone-rocky soils, which are frequently subject to rapid water loss and drought induced by heat stress, and it is difficult for plants to acquire the necessary resources for fast growth. Therefore, the observed influence of N limitation on genome size in *Primulina* cannot be explained by the hypothesis of reallocation of N from DNA to RNA synthesis.

We propose an alternative mechanism for genome size evolution in *Primulina* under N-limitation: competition for nitrogen nutrient between DNA synthesis and cellular functions. Nitrogen is critically important for plants because it is needed in relatively large quantities not only for growth but also for other functions, such as storage, defense, and mechanisms of stress resistance. Recent studies have found that N limitation has caused quantitative and qualitative shifts in the composition of amino acids^35^,^36^, providing evidence that plants are able to mobilize N to optimize N metabolism in response to N limitation. Glutamic acid (Glu), which plays a major role in DNA synthesis and as a donor for the synthesis of other amino acids, was found to be significantly reduced under low-N conditions^36^. Similarly, nitrogen partitioning in oaks revealed both an increase in leaf soluble protein and free amino acid concentrations at the expense of N used for structural components^37^. It seems that there may be a tradeoff between investment of N in amino acids for plant defense functions and amino acids used for DNA synthesis as an adaptive evolutionary strategy under abiotic stress. N is also an essential component for many classes of metabolites such as alkaloids, cyanogenic glucosides and non-protein amino acids. All of them have been reported to accumulate in plants suffering environmental stresses^38^,^39^. In addition, plants normally produce a large variety of phenolic compounds as responses to abiotic and biotic stressors. Increasing stress or decreasing nutrient availability should result in higher production of phenolic compounds, through the competition between protein and phenolic synthesis for their main common precursor, phenylalanine (Phe)^40^. Thus, N limitation should be a key factor linking the competition between DNA synthesis and amino acids, protein and secondary metabolites, because of N-derived precursors. We believe *Primulina,* with its smaller genome size in oligotrophic karst habitats, would be subject to N reallocation to amino acids and/or secondary metabolites as an adaptive response. However, the hypothesis should be further tested through quantifying N partitioning among DNA/RNA, protein, amino acids and secondary metabolites.

## Methods

### Sample collection

The plant sampling has been described in a previous study on genome size and specific leaf area (SLA)^27^. Briefly, we designated populations as the sampling units, for each population, five adult plants were randomly excavated for element analysis. In total, we used materials of 100 populations of 99 species of *Primulina* (Fig. S1) collected for a previous analysis of genome size and SLA^27^. In this study, chemical analyses were performed on dried leaves previously used for SLA calculation. For each species, one population was chosen with the exception for *P. eburnea*, the most widely distributed species that separated into two clades in the phylogeny, and therefore two populations for this species were used. Since most species are narrow endemics and commonly found in single-site limestone karst habitats, we believe such a sampling design adequately reflects variation within the species.

### Chemical analyses

Leaves of the five individuals were washed with double-distilled water and dried at 60°C to constant weight, then ground to a fine powder (2-mm-mesh pore size) and stored in plastic vials for later analyses. Concentrations of leaf C and N were analyzed using a PDZ Europa ANCA-GSL elemental analyzer interfaced to a PDZ Europa 20-20 isotope ratio mass spectrometer (Sercon Ltd., Cheshire, UK). All the analyses were carried out at the University of California (Davis) stable isotope facility. The total P concentrations in leaves were determined via inductively coupled plasma optical emission spectrometry (ICP-OES) following digestion in Teflon beakers with a mixture of ultrapure concentrated HNO_3_ + HClO_4_ (4:1 v/v). The solutions were evaporated to dryness, redissolved in HNO_3_, diluted with 50 mL of MilliQ water and then filtered through a 0.45-mm cellulose acetate membrane (Millipore, Billerica, MA, USA) for analysis. The precision and accuracy of the measurements were controlled through repeated analysis of National Standard Reference materials consisting of poplar leaves (GBW 07604) and agricultural soils (GBW E 070045), respectively.

### Data analysis

The descriptive statistics of leaf elements and their ratios were conducted using JMP pro 10 (SAS Institute Inc., Cary, NC). Based on the uniformity in chromosome numbers in the genus, we interpreted 2C DNA content as genome size throughout the article. Throughout this study, genome size data (2C DNA content) of *Primulina* species were taken from a previous study^27^. The analysis of phylogenetic signal of leaf traits was based on a previously published phylogenetic tree of 104 species of *Primulina*^27^. In this study, we treated all variables as continuous traits. Pagel’s λ^41^ measures the phylogenetic signals in relation to leaf traits. The parameter λ assesses the contribution of phylogeny to the covariance among species for a given trait. If there is no phylogenetic signal, λ will be close to zero, indicating that trait evolution has proceeded independent of the phylogeny. If λ = 1, then the trait has exactly the amount of signal expected for the phylogenetic tree employed and a model of evolution based on a random walk (Brownian motion). Intermediate values of 0 < λ < 1 indicate different degrees of phylogenetic signals. The analyses were carried out using species means with the R package ‘caper’^42^. For each analysis, the significance of the phylogenetic signal was assessed by estimating the likelihood under λ = 0 and λ = 1 via likelihood ratio tests (LRT)^43^.

We examined the relationships between genome size and leaf nutrient concentrations using a phylogenetic generalized least-squares (PGLS) approach^44^, in which phylogenetic regression was performed with a phylogenetic tree whose internal branches were all multiplied by λ, leaving the tip branches at their original length. In this approach, when λ is forced to 0, it is equivalent to ordinary (nonphylogenetic) least-squares regression (OLS), which assumes a star –shaped phylogeny in which residual variation is independent among species. In this study, the R package ‘caper’^42^ was used to compute both types of regression models with λ forced to equal 0 and estimated values, with genome size as dependent variables and leaf nutrient concentrations as independent variable. Likelihood ratio tests (LRT) were used to assess which model had the best fit.

To explore the influence of environmental conditions on leaf nutrients, PGLS was used to assess the relationship between leaf concentrations of N and P and a suit of factors of climatic variables. We extracted 19 bioclimatic variables for average georeferenced records per species from the WorldClim climate database (at 2.5 min scale; http://worldclim.org/bioclim)^45^. These bioclimatic variables represent summaries of temperature and precipitation dimensions of environment.

## Additional Information

**How to cite this article**: Kang, M. *et al.* Nitrogen limitation as a driver of genome size evolution in a group of karst plants. *Sci. Rep.* **5**, 11636; doi: 10.1038/srep11636 (2015).

## Supplementary Material

Supplementary Information

## Figures and Tables

**Figure 1:**
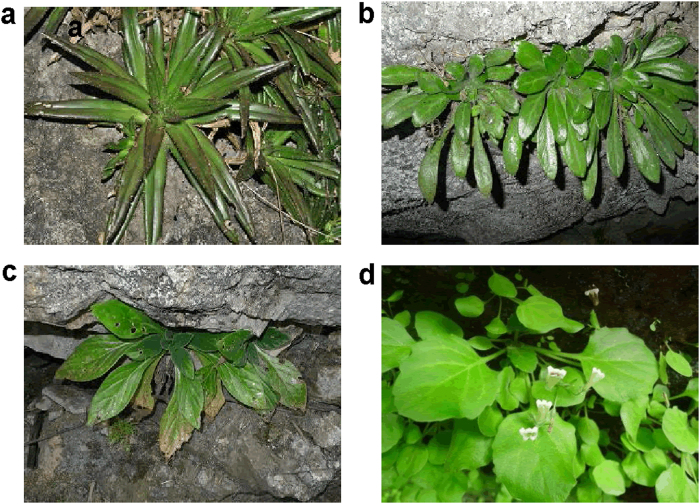
Representative *Primulina* species growing on limestone karsts with poor soil development. The order (**a**–**d**) shows a gradient of increased genome size: (**a**) *P. spinulosa* (2C = 1.47 pg); (**b**) *P. yangchunensis* (2C = 1.76 pg); (**c**) *P. eburnea* (2C = 2.03 pg) and (**d**) *P. xiuningensis* (2C = 2.39 pg).

**Table 1:** Statistics summary of leaf nutrient contents (C, N, P) and C : N : P ratio of 99 *Primulina* species analyzed in this study.

	**Mean**	**SD**	**CV**	**Minimum**	**Maximum**	**λ**	***P***
C (mg g^−1^)	357.97	18.39	0.05	315.43	425.66	0.137	0.556
N (mg g^−1^)	14.65	5.78	0.39	5.38	38.38	**0.759**	**<0.0001**
P (mg g^−1^)	1.45	0.78	0.53	0.46	5.12	**0.234**	**0.048**
C : N	28.18	11.08	0.39	8.72	66.77	**0.776**	**<0.0001**
N : P	11.46	5.09	0.44	3.82	41.24	0	1
C : P	308.11	142.74	0.46	74.28	772.93	**0.295**	**0.018**

Mean, standard deviation (SD), coefficient of variation (CV), minimum and maximum of leaf element concentrations (mg g^−1^ dry mass), Pagel’s λ and probability (*P*) testing for phylogenetic signal. Statistically significant values are in bold (*P* < 0.05).
