# Supplementary material for: Nitrogen limitation as a driver of genome size evolution in a group of karst plants
Source: Sci Rep. 2015 Jun 25;5:11636. doi: 10.1038/srep11636 (PMC4479984; doi:10.1038/srep11636)

Supporting information

Nitrogen limitation as a driver of genome size evolution in a group of karst plants

Ming Kang, Jing Wang, Hongwen Huang

Key Laboratory of Plant Resources Conservation and Sustainable Utilization, South China Botanical Garden, Chinese Academy of Sciences, Guangzhou, China

Table S1 Raw data of the 100 populations of *Primulina* in this study

Species	Genome size (pg)	C (mg g^{-1})	N (mg g^{-1})	P (mg g^{-1})	C : N	N : P	C : P
P. baishouensis	1.78	358.31	14.17	0.94	25.29	15.11	381.99
P. bicolor	2.12	367.36	13.63	1.76	26.95	7.74	208.73
P. bipinnatifida	1.79	425.66	11.84	1.06	35.95	11.17	401.57
P. brachytricha	1.73	381.19	14.97	1.88	25.46	7.96	202.76
P. bullata	1.89	358.59	10.33	0.62	34.71	16.66	578.37
P. cavicola	2.14	341.40	13.11	0.92	26.04	14.25	371.09
P. chizhouensis	1.89	354.94	17.20	1.32	20.64	13.03	268.89
P. cordifoli	2.05	352.79	19.66	1.07	17.94	18.37	329.71
P. cordifolia	2.04	373.01	16.33	2.78	22.84	5.87	134.18
P. danxiaensis	2.35	380.16	31.74	5.12	11.98	6.20	74.28
P. depressa	2.07	360.69	16.29	3.07	22.14	5.31	117.49
P. dongguanica	1.87	353.06	16.54	1.42	21.35	11.65	248.63
P. eburnea.1	1.73	373.54	18.31	1.13	20.40	16.20	330.57
P. eburnea.2	2.03	331.11	13.34	3.49	24.82	3.82	94.87
P. fimbrisepala	2.15	404.14	18.34	1.69	22.04	10.85	239.14
P. glandulosa	2.14	362.96	20.77	1.07	17.48	19.41	339.21
P. gueilinensis	1.96	366.68	13.92	1.41	26.34	9.87	260.06
P. gueilinensis var. brachycarpa	2.54	325.44	27.62	2.32	11.78	11.91	140.34
P. guihaiensis	2.05	346.79	17.55	2.09	19.76	8.41	166.25
P. hedyotidea	1.56	342.23	6.51	1.05	52.57	6.22	326.87
P. heterotricha	1.39	352.54	14.98	2.49	23.53	6.02	141.58
P. hochiensis	1.77	363.80	17.02	1.86	21.37	9.15	195.59
P. huaijiensis	1.12	359.24	5.38	1.01	66.77	5.32	355.33
P. juliae	2.51	352.60	26.97	1.31	13.07	20.64	269.78
P. langshanica	1.80	341.06	12.51	1.87	27.26	6.69	182.39
P. latinervis	2.28	355.38	17.36	2.11	20.47	8.23	168.43
P. laxiflora	1.36	373.85	10.38	0.86	36.02	12.07	434.71
P. leiophylla	1.92	332.41	9.38	0.55	35.44	17.05	604.38

P. lepingensis	2.23	342.52	22.23	3.45	15.41	6.44	99.28
P. leprosa	2.07	345.48	8.66	0.74	39.89	11.70	466.86
P. liboensis	1.98	355.55	7.43	0.46	47.85	16.15	772.93
P. liguliformis	1.68	338.47	11.44	1.21	29.59	9.45	279.73
P. lijiangensis	2.17	370.61	10.46	1.75	35.43	5.99	212.26
P. linearicalyx	1.49	358.38	10.93	0.93	32.79	11.75	385.35
P. linearifolia	1.18	357.57	9.25	1.24	38.66	7.46	288.36
P. lobulata	1.94	377.48	13.70	0.78	27.55	17.56	483.95
P. longgangensis	1.39	366.70	8.86	0.50	41.39	17.72	733.40
P. longicalyx	2.06	354.74	14.81	1.24	23.95	11.94	286.08
P. longii	2.06	363.95	10.45	0.88	34.83	11.88	413.58
P. longistyla	2.16	393.04	20.18	1.18	19.48	17.12	333.37
P. lunglinensis	2.12	347.85	15.56	2.24	22.36	6.94	155.08
P. lungzhouensis	2.07	374.33	10.46	1.28	35.79	8.17	292.45
P. luochengensis	1.32	339.68	10.13	0.50	33.53	20.10	673.97
P. lutea	2.26	369.32	13.39	1.47	27.58	9.11	251.24
P. mabaensis	2.30	337.28	20.55	1.64	16.41	12.53	205.66
P. macrodonta	1.80	367.47	17.90	2.15	20.53	8.33	170.92
P. medica	2.03	358.41	12.01	0.74	29.84	16.34	487.63
P. moii	2.03	380.71	17.40	2.03	21.88	8.57	187.54
P. mollifolia	2.17	359.91	9.78	0.68	36.80	14.45	531.62
P. nandanensis	1.94	353.98	16.83	1.76	21.03	9.56	201.13
P. napoensis	1.90	348.85	11.28	0.98	30.93	11.51	355.97
P. obtusidentata	2.28	394.19	20.91	1.77	18.85	11.81	222.71
P. ophiopogoides	1.38	386.08	7.42	1.01	52.03	7.35	382.26
P. orthandra	1.84	349.05	8.09	1.06	43.15	7.63	329.29
P. parvifolia	1.47	360.68	12.80	0.77	28.18	16.62	468.42
P. pinnatifida	2.28	385.81	17.74	1.12	21.75	15.84	344.47
P. pseudoeburnea	1.72	350.99	11.97	1.61	29.32	7.43	218.01
P. pterppoda	1.46	322.96	15.04	2.03	21.47	7.41	159.09
P. pulchurfolia	1.86	348.09	9.04	1.64	38.51	5.50	211.86
P. qingyuanensis	1.97	350.19	12.14	1.10	28.85	11.00	317.20
P. renifolia	1.66	342.35	13.05	1.05	26.23	12.43	326.05
P. repanda	2.09	388.78	21.54	1.50	18.05	14.36	259.19
P. repanda var. guilinensis	1.95	353.34	13.35	1.63	26.47	8.19	216.77
P. ronganensis	1.94	334.57	14.44	1.18	23.17	12.24	283.53
P. rongshuiensis	1.96	341.92	10.97	1.14	31.17	9.62	299.93
P. roseo-alba	2.03	361.10	14.39	1.42	25.09	10.13	254.30
P. sclerophylla	1.74	335.03	18.64	1.27	17.97	14.68	263.80
P. shouchengensis	2.07	362.10	12.46	1.86	29.06	6.70	194.68
P. sinensis	2.35	367.16	23.80	1.56	15.43	15.29	235.81
P. spinulosa	1.47	365.64	9.39	0.78	38.94	12.04	468.77
P. subrhomboidea	1.87	358.38	20.89	2.29	17.16	9.12	156.50
P. subrhomboidea var. tribracteata	1.97	365.74	6.99	1.21	52.32	5.78	302.26

P. subulata	1.80	346.85	11.45	0.94	30.29	12.18	368.99
P. swinglei	1.62	325.22	15.34	1.28	21.20	11.98	254.08
P. tabacum	1.81	366.38	16.51	1.71	22.19	9.65	214.26
P. tenuifolia	2.10	331.91	18.94	1.77	17.52	10.70	187.52
P. tenuituba	2.15	381.30	12.47	1.50	30.58	8.30	253.69
P. tiandengensis	1.71	353.70	12.94	1.12	27.33	11.55	315.80
P. tribracteata	2.13	367.39	14.20	0.66	25.87	21.48	555.81
P. varicolor	2.01	358.84	8.04	0.96	44.63	8.38	373.79
P. verecunda	1.79	400.37	16.66	1.10	24.03	15.15	363.97
P. villosissima	2.04	338.85	11.26	1.33	30.09	8.47	254.77
P. wentsaii	1.63	350.79	9.05	0.84	38.76	10.77	417.61
P. xiuningensis	2.39	334.77	38.38	4.32	8.72	8.88	77.49
P. xiziae	1.87	350.24	18.73	1.20	18.70	15.57	291.14
P. yangchunensis	1.71	375.54	6.72	0.83	55.88	8.11	453.00
P. yongxingensis	2.53	350.82	31.96	0.78	10.98	41.24	452.67
P. yungfuensis	2.06	344.89	14.19	0.65	24.31	21.76	528.97
P. sp.nov.1	1.85	356.29	10.34	2.41	34.46	4.29	147.84
P. sp.nov.2	1.88	315.43	8.36	0.53	37.73	15.77	595.15
P. sp.nov.3	2.04	355.62	20.75	2.36	17.14	8.79	150.69
P. sp.nov.4	1.89	363.84	14.99	1.22	24.27	12.29	298.23
P. sp.nov.5	1.29	355.04	6.02	0.66	58.98	9.12	537.94
P. sp.nov.6	2.00	362.70	13.24	0.95	27.39	13.94	381.79
P. sp.nov.7	1.86	354.47	9.00	1.27	39.39	7.09	279.11
P. sp.nov.8	1.88	386.30	20.25	1.65	19.08	12.27	234.12
P. sp.nov.9	2.19	339.17	17.79	2.05	19.07	8.68	165.45
P. sp.nov.10	2.17	351.01	15.06	1.27	23.31	11.86	276.39
P. sp.nov.11	2.30	338.11	20.12	1.63	16.80	12.34	207.43
P. sp.nov.12	1.72	363.80	7.02	0.86	51.82	8.16	423.02

Table S2 Summary of genome size (2C DNA content) in relation to leaf nutrient contents under regression models of ordinary least squares (OLS) and phylogenetic generalized least squares (PGLS). The values of log-likelihood (Lh), slope, R^2 and P are indicated. Statistically significant relationships are in bold (P<0.05)

	PGLS				OLS			
	Lh	Slope	R^2	P	Lh	Slope	R^2	P
C (mg g^{-1})	12.992	-0.104	0.002	0.790	-14.659	0.170	0.003	0.734
N (mg g^{-1})	18.784	0.111	**0.120**	**7.7×10^{-6}**	4.707	0.233	**0.299**	**7.84×10^{-14}**
P (mg g^{-1})	14.147	0.034	0.020	0.135	-6.269	0.104	**0.096**	**7.75×10^{-5}**
C: N	17.125	-0.115	**0.098**	**6.7×10^{-5}**	1.733	-0.229	**0.290**	**2.03×10^{-13}**
C: P	14.723	-0.035	0.022	0.144	-6.324	-0.102	**0.092**	**0.0001**
N: P	13.017	0.009	0.002	0.857	-14.587	0.057	0.020	0.137

Table S3 Summary of leaf N concentration in relation to latitude and bioclimatic variables under the regression model of phylogenetic generalized least squares (PGLS)

	Slope	R^2	P
Latitude (°N)	2.14	**0.171**	**4.67×10^{-8}**
BIO 1 = Annual mean temperature	-1.021	**0.096**	**8.28×10^{-5}**
BIO 2 = Mean diurnal range (mean of monthly (max temp - min temp))	-0.317	0.004	0.704
BIO 3 = Isothermality (BIO2/BIO7) (*100)	-1.16	**0.135**	**1.6×10^{-6}**
BIO 4 = Temperature seasonality (standard deviation *100)	0.828	**0.122**	**5.9×10^{-6}**
BIO 5 = Max temperature of warmest month	0.21	0.001	0.907
BIO 6 = Min temperature of coldest month	-0.256	**0.141**	**1.4×10^{-6}**
BIO 7 = Temperature annual range (BIO5-BIO6)	1.217	**0.128**	**3.6×10^{-6}**
BIO 8 = Mean temperature of wettest quarter	-1.21	**0.08**	**0.0003**
BIO 9 = Mean temperature of driest quarter	-0.35	**0.092**	**0.0001**
BIO 10 = Mean temperature of warmest quarter	-0.348	0.003	0.718
BIO 11 = Mean temperature of coldest quarter	-0.424	**0.131**	**2.6×10^{-6}**
BIO 12 = Annual precipitation	0.001	0.0001	1
BIO 13 = Precipitation of wettest month	-0.291	0.019	0.154
BIO 14 = Precipitation of driest month	0.259	**0.056**	**0.004**
BIO 15 = Precipitation seasonality (coefficient of variation)	-0.021	**0.133**	**2.1×10^{-6}**
BIO 16 = Precipitation of wettest quarter	-0.353	0.024	0.094
BIO 17 = Precipitation of driest quarter	0.0275	**0.053**	**0.006**
BIO 18 = Precipitation of warmest quarter	-0.554	**0.078**	**0.0005**
BIO 19 = Precipitation of coldest quarter	0.188	**0.037**	**0.026**

Fig. S1 Sampling locations of the 100 populations representing 99 species of *Primulina* in south China. The map was made with Natural Earth (Free vector and raster map data @ naturalearthdata.com).

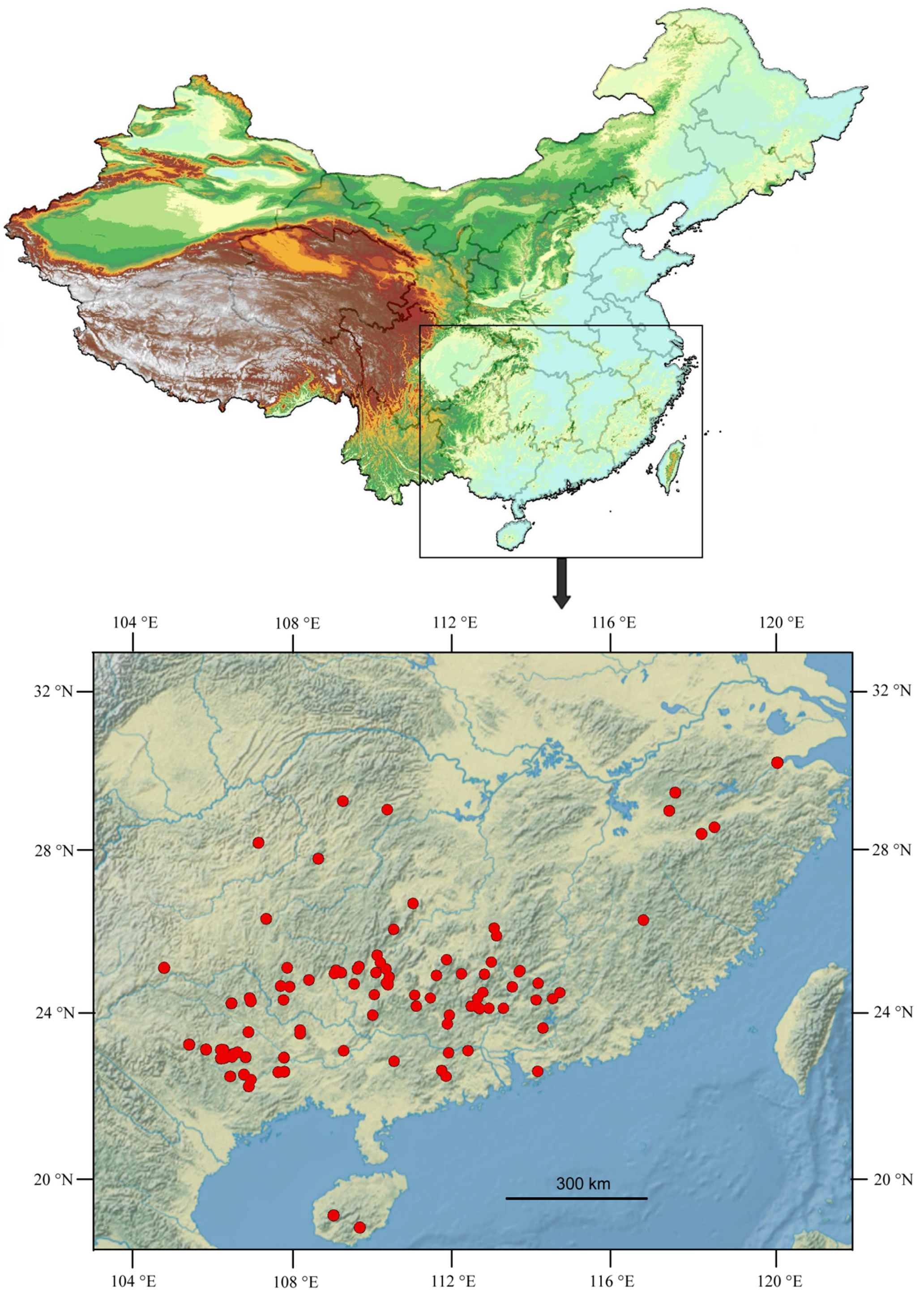

Supplement: Supplementary Information [file srep11636-s1.pdf]
